# Ectopic expression of the *AtCDF1* transcription factor in potato enhances tuber starch and amino acid contents and yield under open field conditions

**DOI:** 10.3389/fpls.2023.1010669

**Published:** 2023-03-01

**Authors:** Laura Carrillo, Edurne Baroja-Fernández, Begoña Renau-Morata, Francisco J. Muñoz, Javier Canales, Sergio Ciordia, Lu Yang, Ángela María Sánchez-López, Sergio G. Nebauer, Mar G. Ceballos, Jesús Vicente-Carbajosa, Rosa V. Molina, Javier Pozueta-Romero, Joaquín Medina

**Affiliations:** ^1^ Centro de Biotecnología y Genómica de Plantas (CBGP) UPM-INIA/CSIC, Campus de Montegancedo, Madrid, Spain; ^2^ Instituto de Agrobiotecnología (IdAB), CSIC-Gobierno de Navarra, Mutiloabeti, Nafarroa, Spain; ^3^ Departamento de Biología Vegetal, Universitat de València. Vicent Andrés Estellés, Burjassot, Spain; ^4^ Instituto de Bioquímica y Microbiología, Facultad de Ciencias, Universidad Austral de Chile, Valdivia, Chile; ^5^ ANID–Millennium Science Initiative Program, Millennium Institute for Integrative Biology (iBio), Santiago, Chile; ^6^ Unidad Proteomica (CNB), Centro Nacional de Biotecnología (CNB-CSIC), Cantoblanco, Madrid, Spain; ^7^ Departamento de Producción Vegetal, Universitat Politècnica de València., València, Spain; ^8^ Institute for Mediterranean and Subtropical Horticulture “La Mayora” (IHSM), CSIC-UMA, Málaga, Spain

**Keywords:** potato, CDF, crop yield, photoassimilate partition, amino acid, starch, C/N metabolism

## Abstract

**Introduction:**

Cycling Dof transcription factors (CDFs) have been involved in different aspects of plant growth and development. In Arabidopsis and tomato, one member of this family (CDF1) has recently been associated with the regulation of primary metabolism and abiotic stress responses, but their roles in crop production under open field conditions remain unknown.

**Methods:**

In this study, we compared the growth, and tuber yield and composition of plants ectopically expressing the *CDF1* gene from Arabidopsis under the control of the 35S promoter with wild-type (WT) potato plants cultured in growth chamber and open field conditions.

**Results:**

In growth chambers, the *35S::AtCDF1* plants showed a greater tuber yield than the WT by increasing the biomass partition for tuber development. Under field conditions, the ectopic expression of CDF1 also promoted the sink strength of the tubers, since 35S::*AtCDF1* plants exhibited significant increases in tuber size and weight resulting in higher tuber yield. A metabolomic analysis revealed that tubers of *35S::AtCDF1* plants cultured under open field conditions accumulated higher levels of glucose, starch and amino acids than WT tubers. A comparative proteomic analysis of tubers of *35S::AtCDF1* and WT plants cultured under open field conditions revealed that these changes can be accounted for changes in the expression of proteins involved in energy production and different aspects of C and N metabolism.

**Discussion:**

The results from this study advance our collective understanding of the role of CDFs and are of great interest for the purposes of improving the yield and breeding of crop plants.

## Introduction

Potato is now the fourth most important food crop worldwide, after wheat, rice and corn and the major staple food in many developing countries (FAO, 2020). In addition, the potato is also grown for processed food, starch production or animal feed. Their tubers are rich in starch (60-80% of the dry matter) as well as quality proteins, vitamins, micronutrients and antioxidants (Burgos et al., 2020). As a major food crop, the potato has a key role in the 2030 agenda for sustainable development, and is vital if the goals aimed at ensuring nutritious and sufficient food are to be met (www.faostat.fao.org).

In the current context of climate change, where a substantial decline in potato yield has been predicted (Haverkort and Struik, 2015; Dahal et al., 2019), improving the production and nutritional quality of tubers is key if agricultural development it to take place(Hameed et al., 2018). Therefore, despite the rise in global production scale during the last few decades, increasing the yield per hectare is a crucial breeding objective in the case of potato (Bradshaw, 2021). The aim behind conventional breeding techniques has been directed to increase yield, processing and storage quality, as well as to enhance disease resistance (Birch et al., 2012). Although these strategies have been successfully employed for the introduction of some traits, such as the resistance to fungal pathogens (Naess et al., 2000; Tek et al., 2004; Halterman et al., 2016), the progress is slow, and it is a challenging task due to tetraploidy, heterozygosity and a lack of variability as regards the traits of interest in commercial cultivars (Patil et al., 2016). Wild species represent a valuable germplasm source, but some wild and cultivated species exhibit sexual incompatibility (Halterman et al., 2016). In this context, during the last few years, the efforts to improve this crop have focused on the development of biotech potato varieties (Barrell et al., 2013). In addition to the improvement of tolerance to pathogens (Martínez-Prada et al., 2021), several analyses succeeded in incorporating nutritional traits in potato, such as enhanced protein content (Chakraborty et al., 2010) and increased levels of some essential amino acids (Dancs et al., 2008; Kumar and Jander, 2017), vitamin C (Bulley et al., 2012), β-carotene (Li et al., 2012) or triacylglycerols (Hofvander et al., 2016), as well as a higher amylose content (Krunic et al., 2018). Furthermore, the reduction of anti-nutrient contents in potato, such as steroidal glycoalkaloids, has also been addressed (Itkin et al., 2013). It is noteworthy that in some of these analyses (Dancs et al., 2008), yield reductions of up to 60% have been described. Only a few reports have shown that genetic engineering can produce transgenic events with an improved tuber yield greater than that produced by conventional breeding under field conditions (Baroja-Fernández et al., 2009; Pourazari et al., 2018). As pointed out by Pourazari et al. (2018), breeding for improved crop quality traits can affect non-target traits related to growth and resource use, and it is important to take into account that these effects may vary in different cultivation conditions (e.g., greenhouse vs. field). Therefore, the selection of suitable genomic targets able to change metabolic aspects while increasing the yield under agronomic crop management conditions is a critical prerequisite if the desired goals are to be reached. In this scenario, we have surveyed the use of DOF transcription factors (like CDFs), which has been associated with the regulation of the primary metabolism in other *Solanaceae* species (Renau-Morata et al., 2017; Domínguez-Figueroa et al., 2020) as a new genetic tool to increase crop production and nutritional quality.

Plant-specific DNA binding with one finger (DOF) proteins are a class of transcription factors (TFs) that exhibit a 50 amino acid conserved DNA binding domain that binds to the DNA motif: 5´-T/AAAG-3´ (Yanagisawa and Schmidt, 1999). DOF proteins play different functions in plant growth and development comprising root growth, shoot branching, seed and tuber development, and flowering (Yanagisawa, 2002; Kloosterman et al., 2013; Noguero et al., 2013; Rueda-Lopez et al., 2017). Maize *ZmDOF1* and *ZmDOF2* have also been involved in C/N balance and nitrogen assimilation (Yanagisawa and Sheen, 1998; Yanagisawa, 2004; Peña et al., 2017) by the differential control of the expression of central genes like *pyruvate kinase (PK)*, *phosphoenolpyruvate carboxylase (PEPC)* and *glutamate synthase (GS)*. In fact, the ectopic expression of *ZmDOF1* in both rice and Arabidopsis increased the expression levels of key genes involved in carbon skeleton production (e.g. *C4-PEPC* and *PK1)* and N assimilation (e.g. *GS* and *glutamate synthase, GOGAT*) (Yanagisawa, 2004; Kurai et al., 2011; Peña et al., 2017).

Amongst different DOFs, cycling DOF Factors (CDFs) play key functions in the photoperiodic pathway, modulating flowering time by controlling the expression of *CONSTANS (CO)* and *FLOWERING LOCUS* (*FT)* (Imaizumi et al., 2005; Fornara et al., 2009; Corrales et al., 2014). In long days, CDF protein stability is tightly controlled by a protein complex constituted by the protein encoded by clock gene *GIGANTEA (GI)* (Park et al., 1999) and the blue-light receptor *FLAVIN BINDING KELCH REPEAT F-BOX PROTEIN 1 (FKF1)*, through specific conserved motifs of 10–30 amino acid residues, located in the CDF C-terminal region (Imaizumi et al., 2005; Sawa et al., 2007; Kloosterman et al., 2013). In potato, it has been described that a *StCDF1* is involved in the photoperiodic control of tuberization by modulating the expression of StSP6A mobile tuberization signal in a StGI and StFKF1 dependent manner (Kloosterman et al., 2013; Abelenda et al., 2019). Potato plants expressing the alleles *StCDF1.2* and *StCDF1.3*, which code for truncated forms lacking the StFKF1 binding domain, are long-day dependent for tuberization, in sharp contrast, genotypes expressing the allele *StCDF1.1* encoding a full-length protein are strictly short-day dependent for tuberization (Kloosterman et al., 2013).

CDFs have also been associated with abiotic stress responses (Fornara et al., 2009; Corrales et al., 2017; Renau-Morata et al., 2017; Renau-Morata et al., 2020a). Arabidopsis *AtCDFs* and tomato *SlCDFs* genes respond to abiotic stresses including dehydration, and osmotic, heat and cold stress (Corrales et al., 2014; Corrales et al., 2017). The ectopic expression of tomato *SlCDF1* and *SlCDF3* genes in Arabidopsis improved tolerance to drought and salinity (Corrales et al., 2014). Moreover, the overexpression of *SlCDF3* in tomato enhanced salt tolerance (Renau-Morata et al., 2017). In the case of potato, the *StCDF1* gene and the long non-coding RNA (lncRNA) counterpart (FLORE), are involved in the control of water loss by modulating both stomatal growth and opening control (Ramírez-Gonzales et al., 2021).

Recent studies have provided evidence for the possible involvement of CDFs in the regulation of nitrogen and carbon metabolisms. Network analyses of a group of 2,174 dynamic N-responsive genes allowed the identification of CDF1 as a key TF that is connected with a second layer of TFs in *Arabidopsis* (Varala et al., 2018). Using the TARGET system, these studies showed that the nitrogen master factor NLP7 binds the promoter region of CDF1, suggesting that CDF1 is a component of the regulatory network involved in Nitrate-signalling cascade (Alvarez et al., 2020). In addition, in Arabidopsis and tomato, we showed that the ectopic expression of *AtCDF3*, the closest homolog of *AtCDF1*, promoted significant changes in the primary metabolism in leaves, including increased levels of sucrose, glutamine, asparagine and GABA in leaves (Corrales et al., 2017; Renau-Morata et al., 2017). Moreover, the ectopic expression of *AtCDF3* in tomato modified the fruit´s amino acid and sugar contents and resulted in enhanced yield and nutrient use efficiency (NUE) under greenhouse conditions (Renau-Morata et al., 2017; Domínguez-Figueroa et al., 2020). Additional functions of tomato SlCDFs in carbon partitioning and carbon metabolism have been described (Renau-Morata et al., 2020a). The targeted overexpression of *SlCDF4* gene in the tomato fruit increased fruit size and total plant yield and promoted a greater partition of carbon resources to the fruit which was related to more sink strength and an increased activity of the sucrose synthase and starch biosynthetic enzymes involved in the sucrose-to-starch conversion process (Renau-Morata et al., 2020b). The overall data indicated that CDFs play significant roles in plant growth and C and N metabolism, and the partition of photoassimilates to the main developing sinks. Therefore, these genes might be used as tools to improve crop yield.

Herein, we have explored the effects of ectopic *AtCDF1* expression in tuber composition and the yield and growth of potato plants cultured under both growth chamber and open field conditions. Notably, a higher tuber yield was obtained in the *35S::AtCDF1* potato plants under agronomic management field conditions, as also observed in growth chambers. The higher production of tubers could be explained by an increased partition of photoassimilates to the developing tubers, leading to a higher proportion of bigger tubers corresponding to the consumption size-category. In addition, also changes in the contents of starch, sugars and quality amino acids, all of which are related to improved tuber quality, were also observed in metabolite analyses. We also characterized changes at the molecular level promoted by the ectopic expression of *AtCDF1* and showed that the overexpression of *AtCDF1* in potato increases tuber yield and improves tuber composition through specific mechanisms involving proteome resetting. Together, our results confirmed the potential of CDF manipulation in breeding programs for crop yield and quality, since a greater sink strength is promoted in the main harvested sink, irrespective of the type of organ.

## Materials and methods

### Potato transformation and growth conditions

The ORFs of the *AtCDF1* gene (*Arabidopsis thaliana*, Col-0) genes were amplified by PCR using cDNA as a template and cloned into a pROK2 vector under the control of CaMV 35S promoter, and the nopaline synthase gene (*NOS*) terminator. The *Agrobacterium tumefaciens* strain LBA4404, was transformed with the resultant plasmid to transform Desiree variety potato plants following the method described by Kiel et al. (1989) with several modifications. Potato transgenic plants were generated by co-culturing leave explants from 4-week-old plants maintained *in vitro* and *A. tumefaciens* harbouring *AtCDF1* construct. To prepare the explants, the petiole and ¼ of the leaf in immediate proximity to the petiole were discarded. The middle of the remaining leaf was manually wounded in two or three places to permit the entry of Agrobacteria into the cells and intercellular spaces. The explant size was approximately of 1 cm^2^. Twenty independent explants were placed in a Petri dish with 40 mL of MS liquid medium (MS medium with salts and vitamins, 20 gL^-1^ sucrose, 0.5 g L^-1^ MES, pH=5.7) containing 320 μL of an *A. tumefaciens* culture (DO_600 nm_=0,8), and incubated at 22°C and without light for two days. Sixty explants were used for each construction to be transformed. Next, the explants were dried on filter paper and cultured on callus induction medium (CIM; 4.4 g L^-1^ MS salts, 16 g L^-1^ 6-Benzylaminopurine (BAP), 5 mg L^-1^ α-naphthaleneacetic acid (NAA), 7 g L^-1^ agar, 50 mg L^-1^ kanamycin, 250 mg L^-1^ cefotaxime) for 7 days. Subsequentely, the explants were transferred to shoot induction medium (SIM; 4.4 g L^-1^ MS salts, 16 g L^-1^ glucose, 0.5 g L^-1^ MES, 0.02 mg L^-1^ (NAA), 0.02 mg L^-1^ gibberellic acid A_3_ (GA_3_), 2 mg L^-1^ zeatin riboside, 7 g L^-1^ agar, 50 mg L^-1^ kanamycin, 250 mg L^-1^ cefotaxime). During shoot generation, the explants were regularly transferred to a fresh medium in 7-10 days intervals until shoots were regenerated. Individual shoots reaching a length of 15-20 mm were excised and transferred to root induction medium (RIM; 4.4 g L^-1^ MS salts, 16 g L^-1^ glucose, 0.5 g L^-1^ MES, 7 g L^-1^ agar, 50 mg L^-1^ kanamycin, 250 mg L^-1^ cefotaxime). Plantlets with well-developed roots were multiplied in *in vitro* culture or transferred to soil for growth chamber and field experiments.

For the assays in growth chambers, the *AtCDF1* overexpressor and WT potato (cv. Desiree) plants were grown under a long day photoperiod of 16-h light/8-h dark photoperiod and a thermoperiod of 23/18°C (light/dark). Plants were grown in 0.5 L pots were fertilized with half-strength Hoagland n°2 solution (Hoagland and Arnon, 1950) for 45 days, then, plant biomass and photosynthesis parameters were determined.

### RNA extraction and qPCR analyses

Total RNA was extracted from leaves and tubers of 2-week-old and 3-month-old potato plants, respectively, following the protocol of Oñate-Sánchez and Vicente-Carbajosa (2008) and treated with DNase (Roche). Then, cDNA was synthesized using 2 μg of DNA-free RNA and the avian myeloblastosis virus reverse transcriptase and oligo-(dT)15 primers (Promega) according to the manufacturer´s instructions.

The expression level of *AtCDF1* gene in overexpression lines was determined by RT-qPCR. A LightCycler^®^480 System (Roche) was used for real-time PCR (10 s at 95°C, 45 cycles of 95°C for 10 s, 60°C for 20 s min and 72°C for 30 s) using LightCycler^®^ 480 SYBR Green I Master (Roche). The *UBIQUITIN* gene from *S. tuberosum* (*PGSC0003DMG400004605*) was used as reference gene to normalize all measurements. The oligonucleotides used are: 5´-TGCTTCACCGTCTCGTCTTCG-3´and 5´-TCCGGGAGTTTCGTGGCCGT-3´ for *AtCDF1*. 5`-AGAAGGCCGGGTGCGTTCTG-3´ and 5`-ACCGGCTTTGCACATCGCCT-3` for *UBIQUITIN (PGSC0003DMG400004605)*. The relative expression levels of the target genes were calculated using the 2-ΔΔ^CT^ method (Livak and Schmittgen, 2001).

### Measurement of photosynthetic activity

Instantaneous values of the net CO_2_ assimilation rate (A_n_), stomatal conductance (g_s_), substomatal CO_2_ concentration (Ci) and transpiration rate (E) were determined with an LI-6400 infrared gas analyser (LICOR Biosciences, USA). The conditions in the measuring chamber were controlled at a saturating PAR of 1,000 μmol m^-2^ s^-1^, 400 ppm CO_2_ and relative humidity of 60-70%. The attached leaf chamber fluorometer enabled the measurement of the effective quantum yield of PSII (PhiPS2). One measurement per plant was taken on the third or fourth leaf from the apex. Eight to ten different plants of each genotype were used.

### Experiment under field conditions

Field trials were conducted in Tudela (Navarra, Spain) during May and September, 2016. Two independent *35S::AtCDF1* lines (L5 and L6), and WT potato (cv. Desiree) plants were used in the field assays. For agronomic analyses, the plants were randomly distributed in three 50 m^2^ parcels, each one containing 30 plants per line. The, plants were grown in a 35 cm spacing plant-plant (distance) in rows 90-cm apart. After 5 months of growth, at the end of the growing season, the total tuber fresh weight (Kg tubers/ha) and total tuber number (no of tubers/ha) were measured. The tubers were graded into three tuber size classes: 0-25 mm (out grade), 25-40 mm (seed) and greater than 40 mm (consumption) and the tuber fresh weight and number per size were measured. The total yield included all the collected tubers (out grades, greens, mechanically damaged, misshapen) while the commercial yield included only seed and consumption sizes. The standard management practices for potato (fertilization and pest/pathogen control) were conducted.

### Determination of the sugar and protein content, amino acid composition and energy-related parameters

For the measurement of the sugar content, total protein content, amino acid composition and energy-related parameters tubers of 30-50 g FW, were harvested from senescent plants, and used. The uber samples were obtained as described in Baroja-Fernández et al. (2009). Three samples were taken from each tuber, immediately freeze-clamped and finally ground to a fine powder in liquid nitrogen.

Soluble sugars (glucose, fructose and sucrose) were determined by HPLC on an ICS-3000 Dionex system as described by Baroja-Fernández et al. (2003). The protein content was determined by using Bradford assay reagent (Bio-Rad XL-100). The starch content was measured enzymatically with an amyloglucosidase-based UV-test Kit (R-Biopharm).

The amylose content in tuber starch was determined using the iodine-based colorimetric method (Andersson et al., 2006). Adenine nucleotides were extracted using HCLO_4_ and measured by HPLC with a Partisil 10-SAX column according to Bahaji et al. (2015). The measurement of reduced (NADH, NADPH) and oxidized nicotinamide adenine dinucleotides (NAD^+^ and NADP^+^) were measured as described by Queval and Noctor (2007). The amino acids profile was analyzed using HLPC, as described in Loiret et al. (2009).

### Protein digestion for proteomic analyses

For the proteomic analyses tubers of 30-50 g FW, harvested from senescent plants in the field trial, were used. The samples were resuspended in lysis buffer containing 8.4 M urea, 2.4 M thiourea, 5% CHAPS, 5 mM TCEP, and a protease inhibitor cocktail (Merck) and incubated for 15 min on ice. The pellet was homogenized by ultrasonication for 5 min on ultrasonic bath, Branson 2510 (Marshall Scientific), was centrifuged at 20000 × g for 10 min at 4°C, and the supernatant containing the solubilized proteins was used for further analysis. Then, 20 µg of protein was precipitated by methanol/chloroform method and resuspended in 20 µL of multichaotropic sample solution UTT buffer (7 M Urea, 2 M thiourea, 100 mM TEAB; Merck).

The sample was then reduced-alkylated with 2 µL of 50 mM TCEP, pH 8.0, at 37°C for 60 min and 1 µL of 200 mM cysteine-blocking reagent MMTS (SCIEX, Foster City, CA) for 10 min at room temperature. The sample was diluted to 140 µL with 25 mM TEAB and the digestion was initiated by adding 1 µg of Pierce MS-grade trypsin (Thermo-Fisher Scientific Inc.) to each sample in a ratio of 1:20 (w/w) and then incubated at 37°C overnight. The sample digestion was evaporated to dryness in a vacuum concentrator.

### Liquid chromatography and mass spectrometer analysis

The digested sample was cleaned up/desalted using Stage-Tips C18 (Merck) and the peptide concentration was determined by Qubit™ Fluorometric Quantitation (Thermo Fisher Scientific). A 1 µg aliquot of each digested sample was subjected to 1D-nano LC-ESI-MS/MS analysis using a nano liquid chromatography system (Eksigent Technologies nanoLC Ultra 1D plus, SCIEX) coupled to a high-speed TripleTOF 5600 mass spectrometer (SCIEX) with a Nanospray III source. Peptides were separated using a 250 min gradient ranging from 2% to 90% mobile phase B (mobile phase A: 2% acetonitrile, 0.1% formic acid; mobile phase B: 100% acetonitrile, 0.1% formic acid).

The data were acquired using an ion spray voltage floating (ISVF) 2300 V, curtain gas (CUR) 35, interface heater temperature (IHT) 150, ion source gas 1 (GS1) 25, and declustering potential (DP) 100 V. All the data were acquired using information-dependent acquisition (IDA) mode (0.25 s MS survey scan in the mass range of 350–1250 Da and 35 MS/MS scans of 100 ms in the mass range of 100–1800 Da) using Analyst TF 1.7 software (SCIEX, USA).

### Data analysis and quantification

The raw instrument files were processed and exported as mgf files. The proteomics data analysis was performed by using 4 search engines (Mascot Server v.2.6.1, OMSSA, XTandem, and Myrimatch) and a target/decoy database built from sequences in the *S. tuberosum* proteome at Uniprot Knowledgebase.

All the searches were configured with dynamic modifications for pyrrolidone from Q (-17.027 Da) and the oxidation of methionine residues (+15.9949 Da) and static modification such as methylthiolation (+45.987 Da) on cysteine, monoisotopic masses, and trypsin cleavage (max 2 missed cleavages). The peptide precursor mass tolerance was 10 ppm, and the MS/MS tolerance was 0.02 Da. Score distribution models were used to compute peptide-spectrum match p-values (Ramos-Fernández et al., 2008), and the spectra recovered by an FDR* <= 0.01 (peptide-level) filter were selected for quantitative analysis. The differential regulation was measured using linear models (Lopez-Serra et al., 2014), and statistical significance was measured using q-values (FDR, False Discovery Rate). All the analyses were conducted using software from Proteobotics (Madrid, Spain). The data were deposited at ProteomeXchange Consortium PXD036489. The Gene Ontology (GO) enrichment analyses were performed on the up- and down-regulated proteins using gPROFILER software (Reimand et al., 2016). Significantly enriched (Padj < 0.05) GO terms describing a biological process (GO BP) for the differential expressed proteins are displayed shown in the figures. The significant metabolic pathways were analyzed using the Mapman tool (Schwacke et al., 2019) The most over-represented biological functions are shown in **Table S2**.

### Data analysis and statistics

The data sets were subjected to analysis of variance (ANOVA) procedures with the Statgraphics statistical software (Statgraphics Centurion XVI, Statpoint Tech, Inc., USA). The mean values were analyzed (*p* < 0.05 or *p* < 0.01) by a Student-Newman-Keuls test.

## Results

### The ectopic expression of *AtCDF1* increases the potato tuber yield

**Figure 1:**
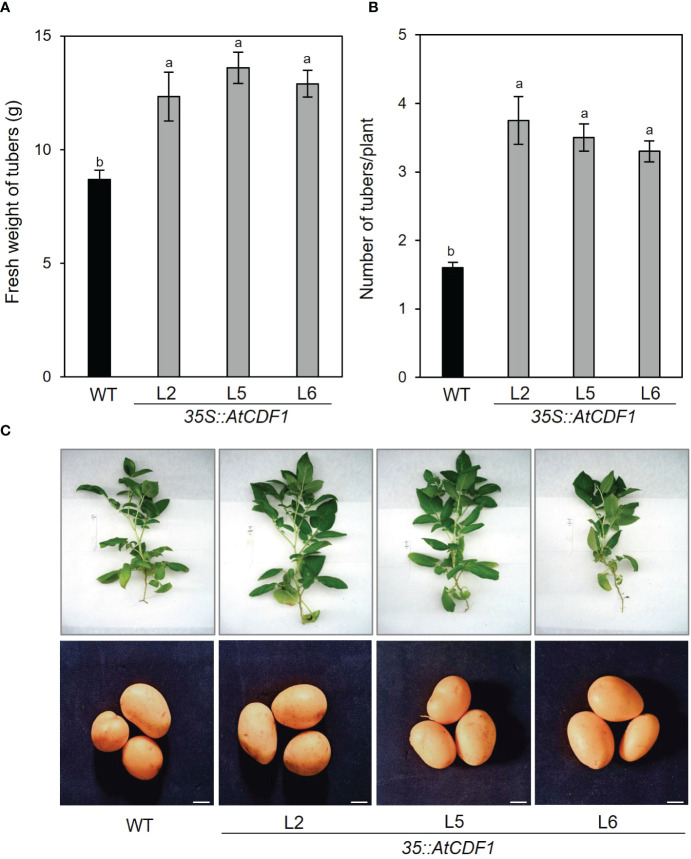
Tuber production of *35S::AtCDF1* and WT potato plants grown under growth chamber conditions. **(A)** Tuber yield (Fresh weight (FW) of tubers/plant) and **(B)** number of tubers per plant of overexpressing *35S::AtCDF1* (L2, L5 and L6 lines) and untransformed control (WT) potato (cv. Desiree) plants grown in growth chambers. **(C)** Representative pictures of plants and tubers of the analyzed lines. Data are expressed as means ± SE of 8 to 10 plants. Different letters above the bars indicate a significant difference for a given genotype between WT and *35S::AtCDF1* plants (*p* < 0.01) according to an analysis of variance, followed by a Student-Newman-Keuls test.

In order to examine the functions of *AtCDF1* and to assess its impact on plant growth and production in potato, a detailed phenotypic characterization *of AtCDF1* gain-of-function plants was conducted by analyzing their performance. Thus, three independent *35S::AtCDF1* lines (L2, L5 and L6) were obtained as described in Materials and Methods. As shown in **Figure S1**, At*CDF1*, transcript levels in leaves and tubers were much higher in all the transgenic *35S::AtCDF1* lines than in the WTPlant biomass and photosynthesis were measured in the *35S::AtCDF1* and WT potato plants after 45 days of growth at 24°C under long-day photoperiod (16h light/8 h dark) in growth chamber conditions. As shown in **Figures 1**, **S1**, **S2**, the transgenic plants and WT showed similar external phenotypes as well as total plant biomass (**Figure S2E**). Accordingly, there were no significant differences photosynthesis activity of WT and *35S::AtCDF1* plants, since similar net photosynthetic rates and effective quantum yields of PSII were observed (**Figures S1A, D**). Notably, *35S::AtCDF1* potato plants (L2, L5 and L6 lines) produced 105-136% more tubers (**Figure 1B**) and a 41-56% higher total tuber yield (**Figure 1A**) per plant than WT plants (*p* < 0.05). The total biomass partitioned to the tubers (estimated as the percentage of total biomass (DW) partitioned in tubers) is consistently 15-25% higher in the *CDF1*-overexpressing plants than in the WT ones (**Figure S2F**), suggesting an increased partition of photoassimilates in tuber development in the transgenic plants.

**Figure 2:**
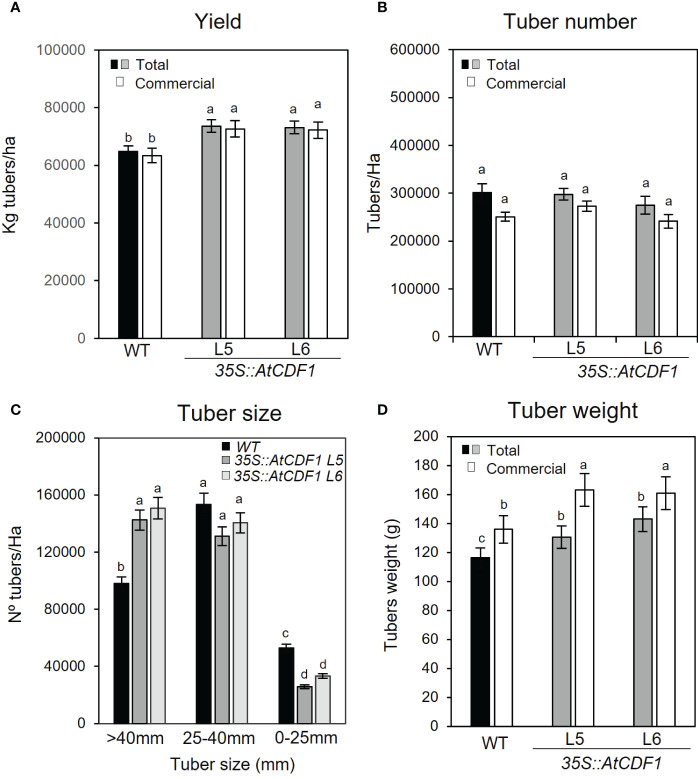
Agronomic performance of *35S::AtCDF1* and *WT potato* plants under field conditions. **(A)** Tuber yield (Kg tubers/ha) and **(B)** tuber number (no tubers/ha) of total and commercial tubers in untransformed control (WT; cv Desiree) and *35S::AtCDF1* (L5 and L6 lines) potato plants. **(C)** Number of tubers in 3 size categories: 0-25 mm (out grade), 25-40 mm (seed) and greater than 40 mm (consumption). **(D)** Mean tuber weight (g FW) of total and commercial tubers. Data are expressed as means ± SE of 10 plants. Scale bar 0.5 cm. Significant differences between WT and *35S::AtCDF1* lines are indicated with different letters (*p* < 0.05) according to an analysis of variance, followed by a Student-Newman-Keuls test.

We also compared the agronomic performance of *35S::AtCDF1* (lines L5 and L6) and WT plants cultured under open field conditions by analysing both the total and the commercial (consumption and seed tubers) plant yield (Kg/ha) and the number of tubers per hectare (no tubers/ha). We found contrasting differences between the yield-related parameters of the transgenic and WT potato plants (**Figure 2**). As illustrated in **Figure 2A**, *35S::AtCDF1* plants produced 13-14% higher total and commercial-size tuber yields than the WT plants (*p* < 0.05). In terms of productivity per land surface unit, control plants produced an average of 64 tonnes of tubers/hectare, whereas *AtCDF1*-overexpressing plants produced about 73 tonnes of tuber/hectare (**Figure 2A**).

Notably, although we did not observed any significant differences (*p* < 0.05) in the number of tubers (n° tubers/ha) between *35S::AtCDF1* and WT plants (**Figure 2B**), we found significant differences (*p* < 0.05) in the size distribution of the tubers (**Figure 2C**). These were graded into three tuber size classes: out grade (0-25 mm), seed (25-40 mm) and consumption (greater than 40 mm). As shown in **Figure 2C**, the overexpressor lines produced 45-53% more tubers of the consumption size grade (**Figure 2C**) than the WT plants. However, it was also noticed that *35S::CDF1* lines showed a reduction of 8-15% and 37-51% in the number of seed and out grade tubers (smaller size categories) than those of the WT plants, respectively (**Figure 2C**). Moreover, the mean tuber weights of the *35S::CDF1* overexpressor plants (L5 and L6 lines), were ca. 12-23% and 17-19% heavier in the total and commercial categories than those of the WT, respectively (*p* < 0.05; **Figure 2D**). Overall these data indicated that under field conditions the agronomic performance of *35S::AtCDF1* plants was better and demonstrated consistent improvement as compared to WT plants. In addition, the higher yield of *35S::AtCDF1* is likely due to an increased partition of biomass to the tubers, allowing the development of a higher number of bigger size tubers of the consumption size grade.

### The ectopic expression of *AtCDF1* promotes starch and amino acid accumulation in the tubers of plants cultured under open field conditions

Previously, we showed that the overexpression of *AtCDF3*, the closest *AtCDF1* homolog, in tomato, modified the amino acid and sugar contents of fruits (major sink organ) and resulted in enhanced yield and NUE under greenhouse conditions (Renau-Morata et al., 2017; Domínguez-Figueroa et al., 2020). These results suggest that CDF3 plays an important role in both N and C assimilation and photoassimilates partition; and thus, CDFs might be used as a biotechnological target for the purposes of improving crop yields.

**Figure 3:**
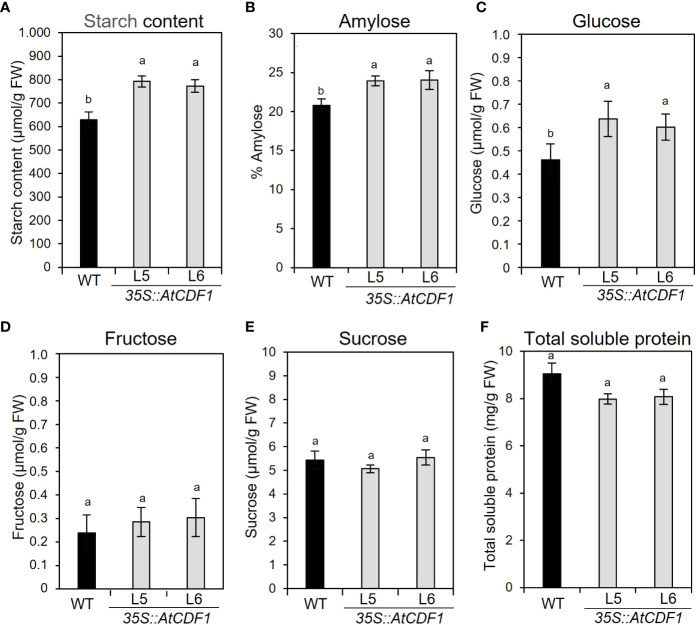
Starch, amylose, soluble sugars and protein contents in tubers of *35S::AtCDF1* and WT plants. **(A)** Starch content, **(B)** amylose percentage in starch, **(C)** glucose, **(D)** fructose and **(E)** sucrose contents, and **(F)** total soluble protein in tubers *35S::AtCDF1* (L5 and L6 lines) and WT (cv. Desiree) plants grown under field conditions. Data are expressed as means ± SE of six replicates. Different letters above the bars indicate a significant differences between WT and *35S::AtCDF1* lines (*p* < 0.05) according to an analysis of variance, followed by a StudentNewman-Keuls test.

**Figure 4:**
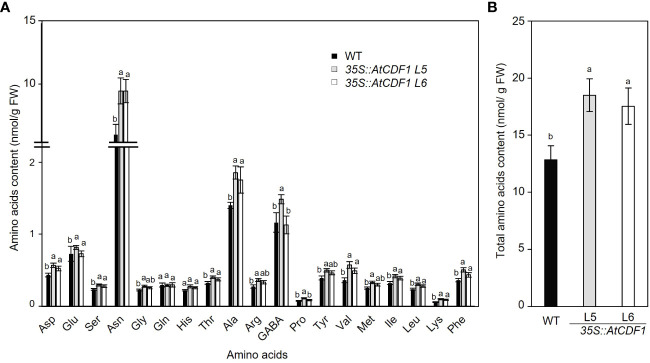
Amino acids contents and composition in tubers of *35S::AtCDF1* and WT potato plants. **(A)** Amino acid composition profile and **(B)** total soluble amino acids in tubers of control (WT; cv Desiree) and *35S::AtCDF1* (L5 and L6 lines) potato plants grown under field conditions. Data are expressed as means ± SE of nine independent replicates per line. For each amino acid, different letters above the bars indicate a significant difference to WT (*p* < 0.01) according to an analysis of variance, followed by a Student-Newman-Keuls test.

To examine the possible effects of *AtCDF1* ectopic expression on the C and N metabolism of potato tubers, as a major sink organ, we compared the contents of soluble sugars, starch and amino acids in tubers of *35S::AtCDF1* plants and WT plants cultured under open field conditions. As shown in **Figure 3**, *35S::AtCDF1* tubers accumulated similar levels of sucrose and fructose, but contained 30-39% higher levels of glucose than the WT tubers. Notably, the starch and amylose contents in *35S::AtCDF1* tubers were ca. 25% and 17% higher than in WT tubers, respectively. These data suggest that CDFs may be upstream regulators of starch biosynthesis pathways likely modulating, directly or indirectly, the activity of key C metabolic proteins. In addition, it has been shown that the tuber can import but also conduct the “*de novo*” biosynthesis of all amino acids (Roessner et al., 2001; Roessner-Tunali et al., 2003). Therefore, we decided to investigate the impact of AtCDF1 on the amino acid content in tubers of At*CDF1* overexpressor and WT plants grown under field conditions. An analysis by GC-MS of the pool sizes of the different amino acids uncovered significant increases in most of the amino acids, especially asparagine, valine, isoleucine and alanine (*p* < 0.05; **Figure 4A**). Consistently, the total amino acid content in the *35S::AtCDF1* tubers were ca. 22-58% higher than in the WT tubers (**Figure 4B**).

**Figure 5:**
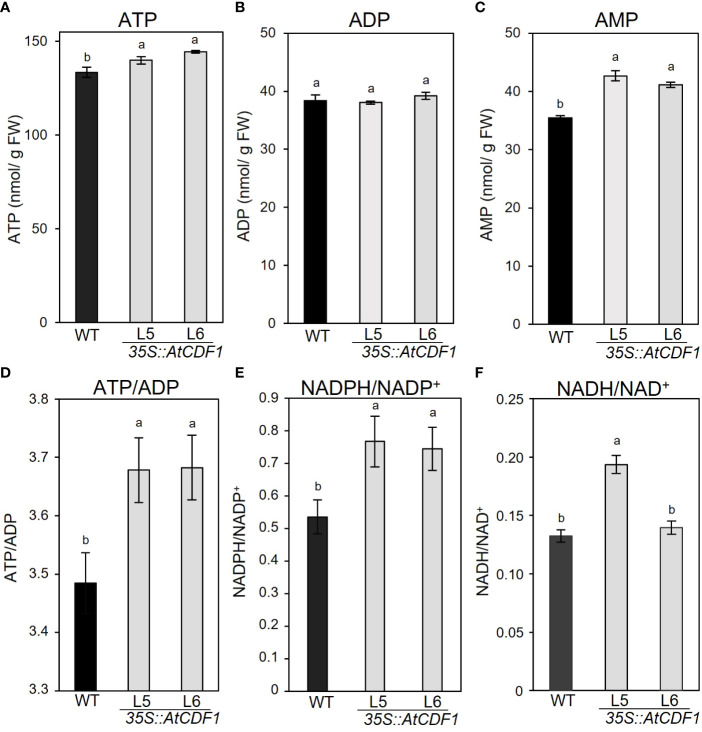
Levels of adenine nucleotides in potato tubers of WT and *35S::AtCDF1* plants. **(A)** ATP, **(B)** ADP, and **(C)** AMP contents, and **(D)** ATP/ADP, **(E)** NADPH/NADP^+^ and **(F)** NADH/NAD^+^ ratios of tubers of control (WT; cv. Desiree) and *35S::AtCDF1* plants (L5 and L6 lines) grown under field conditions. Data are expressed as means ± SE of nine independent replicates per line. Significant differences between WT (cv Desiree) and transgenic plants are indicated with different letters (*p* < 0.05) according to an analysis of variance, followed by a Student-Newman-Keuls test.

It is well known that the levels of adenylate pools are important factors that control/limit the starch content and yield and the concentration of different amino acids (Regierer et al., 2002; Carrari et al., 2005). In order to study the effects of the overexpression of *AtCDF1* on adenylate pools in tubers, we determined the steady-state levels of all three metabolites ATP, ADP, and AMP. The overexpression of At*CDF1* led to significant changes in the levels of the various adenylate pools (**Figure 5**). Notably, the ATP and AMP contents in the *AtCDF1*-overexpressing tubers were ca. 4-5% and 12-16% higher than in ones in the WT tubers; but no significant changes were observed in the ADP levels of the *35S::AtCDF1* lines (**Figure 5**). The ATP/ADP ratio is usually estimated as a central control parameter of cellular energy metabolism that determines the free-energy change for ATP hydrolysis and, therefore, the driving force for many reactions (Atkinson, 1968; Hardie and Hawley, 2001; Geigenberger et al., 2010).Thus we examined the ATP/ADP ratio and identified that were ca. 5-6% higher in tubers of *AtCDF1* overexpression plants than in the ones of the WT (**Figure 5D**), however, ATP/AMP ratio showed no significant changes (**Figure S3**).

We also analysed the content of the NAD(P)H/NAD(P) ratios. An accumulation of evidence has suggested that the nicotinamide adenine dinucleotide NADH/NAD^+^ and NADPH/NADP^+^ redox couples play crucial roles in the regulation of the cellular redox state, energy metabolism, mitochondrial function and gene expression, as well as in signalling pathways (Hashida and Kawai-Yamada, 2019; Smith et al., 2021); therefore this indicated that these redox couples are essential for the maintenance of a wide array of biological processes. Notably, NADPH/NADP^+^ and NADH/NAD^+^ ratios were ca. 39-43% and 1-29% higher in tubers of the *AtCDF1* overexpression plants (L5 and L6 lines) than in the WT (**Figures 5E, F**).

### The ectopic expression of *AtCDF1* in potato promotes significant changes in the tuber proteome

**Figure 6:**
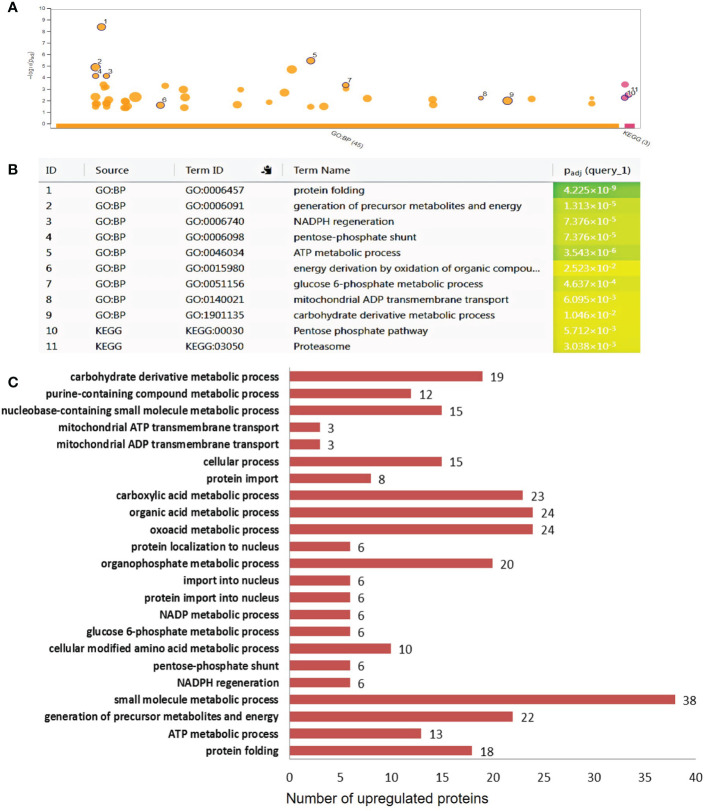
Functional categorization of the differentially upregulated proteins in *35S::AtCDF1* potato tubers. The significantly up regulated proteins as compared with control (WT, cv. Desiree) were organized according to their functional category using to gPROFILER software (Reimand et al., 2016). **(A)** The results of enrichment analysis (GO BF and KEGG) were shown in a Manhattan plot. The x-axis indicates functional terms grouped by database used (color code) and the *y*-axis indicates the enrichment adjusted *p*-values. The dots shows all the enriched terms (*p* < 0.05). The highlighted dots show GO terms emphasized in the text. The graphs **(B, C)** show the enriched terms highlighted in the Manhattan plot as well as the statistical significance (*p-v*alue) and the number of DEPs included in the enriched term (located close to the bar). The full details of the GO analysis are given in **Supplementary Table S2**.

**Figure 7:**
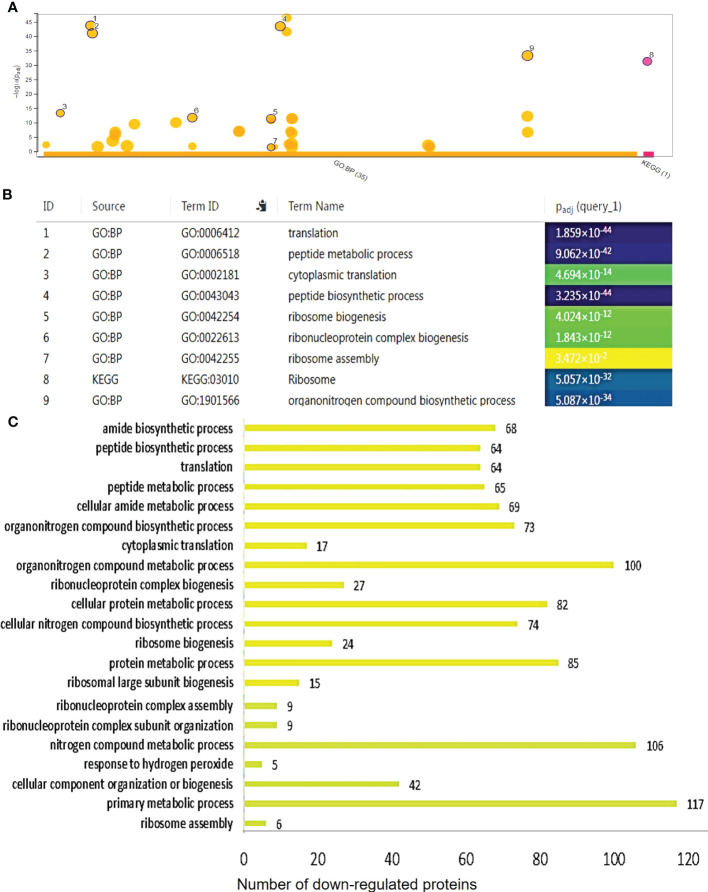
Functional categorization of the differentially downregulated proteins in *35S::AtCDF1* potato tubers. The significantly down-regulated proteins as compared with control (WT; cv. Desiree) were organized according to their functional category using gPROFILER software (Reimand et al., 2016). **(A)** TheResults of enrichment analysis (GO BF and KEGG) were shown in a Manhattan plot. The x-axis indicates the functional terms grouped by the database used (color code), and the y-axis indicates the enrichment adjusted p-values. The dots shows all the enriched terms (*p* < 0.05). The highlighted dots show terms emphasized in the text. The graphs **(B, C)** show the enriched terms highlighted in the Manhattan plot as well as the statistical significance (*p-*value) and the number of DEPs included in the enriched term (placed next to the bar). The full details of the GO analysis are given in **Supplementary Table S2**.

To further investigate the underlying mechanisms related to the observed effects of the ectopic expression of *AtCDF1* on tuber production and tuber quality parameters, we performed a differential proteomic analysis between tubers of *35S::AtCDF1* and WT plants grown under field conditions using LC-ESI MS/MS (deposited at ProteomeXchange Consortium PXD036489). These analyses revealed that the ectopic expression of *AtCDF1* promotes significant changes in the tuber proteome (**Figure S4**). As shown in **Table S1**, of the 407 differentially expressed proteins (DEPs) identified in this study (*q* value < 0.05), 213 were up-regulated and 194 were down-regulated by the ectopic expression of *AtCDF1* (**Figures 6**, **7**).

To identify differentially expressed pathways in *35S::AtCDF1* and WT tubers, a gene ontology (GO) term enrichment analysis according to molecular function (GO MF), biological process (GO BP) and cellular compartment (GO CC) was performed on the differentially expressed proteins. Substantially regulated GO terms are shown in **Figures 6**, **7**. The list of all the GO terms associated with the regulated proteins is provided in **Table S2**. The over-representation analysis of biological functions associated with DEPs significantly up-regulated in *35S::AtCDF1* tubers revealed an enrichment in GO terms related to different aspects of primary metabolism including “ATP metabolic process”, “generation of precursors metabolites and energy”, “NADPH regeneration”, “carbohydrate derivative metabolic process”, “glucose-6-phosphate metabolic process” and “pentose-phosphate pathway” (**Figure 6**; **Table S2**). Specifically, we identified 81 proteins including ADP/ATP mitochondrial carrier proteins, ATP synthase subunits and the enzymes involved in sucrose/starch metabolism and pentose phosphate pathway (e.g. sucrose synthase [SuSy], granule-bound starch synthase [GBSS] 1, SNF1-related protein kinase regulatory subunit gamma-1 [SnRK1], transaldolase and 6-phosphogluconolactonase) (**Table S1**). These analyses also identified the different up-regulated DEPs involved in various aspects of oxidative metabolism, including glycolysis (e.g. pyrophosphate fructose-6-phosphate-1-phosphotransferase, fructose-bisphosphate aldolase, pyruvate kinase and enolase) and TCA cycle (e.g. citrate synthase and malic enzyme), as well as PEP carboxylase (**Table S1**). Moreover, we found GO terms related to nitrogen metabolism and protein folding that are known to be regulated by CDFs (Renau-Morata et al., 2020a). Specifically, we found an enrichment in terms such as “cellular modified amino acid metabolic process” and “protein folding”, including proteins like glutamate decarboxylase (involved in GABA biosynthesis) and betaine aldehyde dehydrogenase, and Heat shock 70, 80 proteins and chaperonins (**Figure 6**; **Tables S1**, **S2**), as well as a group of storage proteins including several patatin proteins (**Table S1**). The GO term analysis of the down-regulated proteins revealed the overrepresentation of terms associated with translation, ribosome biogenesis and protein biosynthesis such as “ribosome assembly”, “ribosome biogenesis”, “translation”, “peptide biosynthetic process” and “ribosome” (**Table S2**). Specifically, we identified a group of downregulated translation- and ribosome biogenesis-related proteins including elongation factors-1-α, -3, eukaryotic translation initiation factor 5A, lysyl/seryl-tRNA synthetases and ribosomal proteins such as L9-1, L13, L26, L27a, S30 and S16 (**Figure 7**; **Table S1**). This analysis also revealed that *35S::AtCDF1* tubers accumulated lower levels of the enzymes involved in starch breakdown (e.g. starch phosphorylase and alpha-amylase) than WT tubers (**Table S1**).

## Discussion

DOF TFs have been extensively explored in various model plants and crops (Yanagisawa, 2016; Renau-Morata et al., 2020a). Of these, CDF TFs are known to be involved in the control of different facets of plant growth and responses to abiotic stresses (Corrales et al., 2014; Fornara et al., 2015; Corrales et al., 2017). In this study, we assessed the impact of the ectopic expression of the Arabidopsis *CDF1* gene on tuber composition and the yield and growth of potato plants cultured under both growth chamber and open field conditions.

### The ectopic expression of *AtCDF1* enhances the tuber yield and quality in potato plants by increasing tuber sink strength

The ectopic expression of *AtCDF1* in potato did not alter the photosynthetic capacity or the total biomass of the plant, but significantly enhanced tuber yield when the plants were cultured in growth chambers (**Figures 1**, **S1**, **S2**). This clearly indicates that CDFs could play an important role in the control of photoassimilates carbon partitioning by increasing tuber sink strength. The data obtained in this study are coherent with previous results pertaining to tomato and Arabidopsis CDFs, since the ectopic expression of *AtCDF3* and *SlCDF3* in tomato promoted higher yield and improved the fruit`s amino acid and sugar contents (Renau-Morata et al., 2017). Moreover, the targeted overexpression of the tomato *SlCDF4* gene in the fruit increased the fruit size and yield, which were related to a greater amount of both water and dry matter accumulated in the fruit (Renau-Morata et al., 2020b).

Under both growth chamber and field conditions, *35S::AtCDF1* lines exhibited significant increases in tuber yield (**Figures 1**, **2**). However, our study revealed differences in the tuber traits depending on the experimental conditions. The higher yield of the overexpressor lines in the growth chambers was related to a higher number of developed tubers. However, the number of tubers in the field were similar in both the WT and *35S::CDF1* plants and the greater yield was due to an increase in both the mean size and weight of the tubers. Similarly, other analyses of potato indicated that the cultivation conditions clearly influence growth-related traits. The tuber yield and the aboveground biomass differ depending on whether they are greenhouse or field conditions (Pourazari et al., 2018). Therefore, due to the marked effect of the environment on targeted and non-targeted traits in GM plants, the performance of the lines has to be verified under commercial management conditions (Glandorf and Breyer, 2016). In line with this presumption, different molecular analyses have demonstrated that *CDF* gene expression is differentially modulated by the environmental conditions (Imaizumi et al., 2005; Sawa et al., 2007; Kloosterman et al., 2013; Corrales et al., 2017; Renau-Morata et al., 2020a).

Notably, here we found that the ectopic expression of *AtCDF1* triggers significant changes in carbon metabolism (**Figures 3**, **6**, **7**). Tubers of plants overexpressing *AtCDF1* accumulated ca. 25% more glucose and starch, thus improving the nutritional value of the tuber for food. In addition, a higher amylose content (ca. 17% compared to WT) was also found in the transgenic tubers (**Figure 3**). Amylose content is a valuable trait associated with different industrial applications of starch. In fact, “high amylose” starches tend to have greater retrogradation rates following processing than those that have a high amylopectin content (Singh and Anderson, 2004). In addition, high amylose starches show high freeze-thaw quality (Jobling et al., 2002) and reduced oil penetration, which are highly valuable tools in the food processing industry (Tarn et al., 2006). Consequently, developing potato cultivars with a high amylose content is an active research area in crop biotechnology (Santelia and Zeeman, 2010; Patil et al., 2016; Dahal et al., 2019);.

The amino acid content is also an important determinant of the tuber`s nutritional and processing quality (Brierley et al., 1996). The ectopic expression of *AtCDF1* promoted significant increases in the contents of essential amino acids (Reeds, 2000), including valine, phenylalanine, threonine, methionine, leucine, isoleucine and lysine in tubers. It is interesting to point out that valine, methionine and leucine are limiting amino acids in the commercial isolated potato protein (Jiménez-Munoz et al., 2022 and references therein). Besides, *35S::AtCDF1* tubers exhibited higher levels of expression of a group of proteins involved in proteolytic pathways (**Figure 6**; **Table S1**). It is, thus, conceivable that *CDF1* mediates the tuber´s amino acid content and nutritional quality through the action on protein turnover and degradation-related pathways. In addition, it is also possible that AtCDF1 might play a role in the control of amino acid translocation from source to sink organs. Interestingly, the *35S::AtCDF1* tubers showed higher levels of patatin proteins than the WT ones (**Table S1**), which are recognized as important nutritional components of potato tubers (Bárta and Bártová, 2009), and have properties such as foaming, emulsifying and enzymatic activities, which are of interest in a number of different biotechnological applications (Ralet and Gueguen, 2001; Van Koningsveld et al., 2006). Overall, the data presented in this study show that the ectopic expression of *AtCDF1* can be used to improve the yield and quality properties and breeding of potato for different industrial applications.

### Overexpression of *AtCDF1* gene results in major changes in the tuber proteome

Up to now, previous studies have mainly used transcriptomic approaches to investigate the molecular mechanism of action of CDFs (Fornara et al., 2009; Yanagisawa, 2016; Corrales et al., 2017; Renau-Morata et al., 2017; Renau-Morata et al., 2020a). However, transcript abundance on its own cannot be utilized to directly deduce directly changes in the proteome and/or metabolic activity/fluxes in the plant metabolism (Nakaminami et al., 2014; Schwender et al., 2014). In this study, we conducted comparative proteomic analysis between *35S::AtCDF1* and WT tubers and identified a group of 407 differentially expressed proteins (DEPs) involved in different aspects of C and N metabolism (**Figures 6**, **7**; **S6**; **Tables S1**, **S2**). Consistent with the above data showing that the *AtCDF1* ectopic expression enhances the potato starch content and yield, these analyses revealed that the ectopic expression of *AtCDF1* upregulated the expression of strong determinants of sink strength, as well as tuber starch content, tuber quality and yield, such as SuSy and GBSS 1 and 2 (Hovenkamp-Hermelink et al., 1987; Baroja-Fernández et al., 2009; Li et al., 2013; Toinga-Villafuerte et al., 2022). In addition, the expression of enzymes involved in starch breakdown, such as starch phosphorylase and alpha-amylase, were downregulated (**Tables S1**, **S2**). This indicates that CDFs act as upstream regulators of C metabolism. In line with this hypothesis, previous reports have shown that the ectopic expressions of Arabidopsis *AtCDF3* and tomato *SlCDF3* in tomato impacts C/N metabolism genes, promoting a rise in sugar and amino acids contents, thus allowing increased growth and yield (Renau-Morata et al., 2017; Domínguez-Figueroa et al., 2020). Moreover, the targeted overexpression of the tomato *SlCDF4* gene in the fruit led to a larger amount of biomass been partitioned to the fruit; this was based on the greater sink strength of the fruits induced by the increased activity of enzymes involved in the sucrose and starch metabolism, including invertase, SuSy, ADP-glucose pyrophosphorylase (AGP) and UDP-glucose pyrophosphorylase (Renau-Morata et al., 2020b).

The proteomic analyses also revealed that the ectopic expression of *AtCDF1* upregulated the expression of SnRK1 and 14-3-3 proteins. The SnRK1 plays an important role in the regulation of development and metabolism in potato tubers and thus yield and quality (McKibbin et al., 2006; Crozet et al., 2014; Wurzinger et al., 2018). 14-3-3 proteins regulate key process in C/N metabolism by direct binding and fine-tuning the activity of a wide array of essential enzymes involved in carbohydrate and nitrogen metabolism, such as nitrate reductase, sucrose phosphate synthase, AGP, glutamine synthetase or H^+^-ATPase (Oecking et al., 1994; Bachmann et al., 1996a; Toroser et al., 1998; Moorhead et al., 1999; Sehnke et al., 2001; Comparot et al., 2003; Chevalier et al., 2009).In addition, the overexpression of different *14-3-3* genes in potato disturbed the sugar, catecholamine and lipid contents. In contrast, a 14-3-3 antisense affected the tuber starch content, nitrate reductase activity and amino acid composition (Prescha et al., 2001; Swiedrych et al., 2002; Zuk et al., 2005). Overall, our data suggest that the ectopic expression of *AtCDF1* affects primary metabolism, at least in part, through SnRK1 and 14‐3‐3 regulatory proteins.

It is well known that the adenylate pools determine the starch content and yield and the accumulation of different amino acids (Tjaden et al., 1998; Loef et al., 2001; Regierer et al., 2002; Carrari et al., 2005). In plant heterotrophic tissues, the energy metabolism and adenylate energy charge are determined by cell respiration pathways (van Dongen et al., 2011). The proteomic analyses revealed that the ectopic expression of *AtCDF1* upregulates the glycolytic and pentose phosphate pathways, the tricarboxylic acid (TCA) cycle and the mitochondrial electron transport chain, as well as adenylate kinases and the ADP-ATP mitochondrial carrier (**Tables S1**, **S2**; **Figure S5**). These data would support the higher ATP content and NAD(P)H/NAD(P)^+^ ratios in *35S::AtCDF1* tubers than in the WT ones (**Figure 5**) which could be channelled for the biosynthesis of starch and amino acids in the tubers.

Overall, these data might suggest that CDF1 may regulate the expression of genes encoding the key enzymes involved in primary metabolism, in all likelhood by direct binding to their promoter regions. The examination of the promoter regions of the highlighted DEPs involved in different processes, such as the ATP metabolism, the enzymes involved in sucrose/starch metabolism, the pentose phosphate pathway, the TCA cycle, as well as the genes involved in amino acid metabolism, ribosome biogenesis and protein folding, consistently showed multiple putative DOF binding sites in their promoter regions (**Figure S6**), suggesting that these groups of genes might be direct targets of CDF1.

In conclusion, the present study showed that the expression of the *CDF1* gene from Arabidopsis increased the yield of potato plants under both chamber and field conditions, and enhanced the tuber quality properties for different industrial applications. The increased yield observed was associated with changes in photoassimilates partitioning and C and N metabolism, as well as with changes in the expression of the proteins involved in energy production and sucrose and starch metabolisms. Together with what was previously described for tomatoes (Renau-Morata et al., 2017; Domínguez-Figueroa et al., 2020; Renau-Morata et al., 2020b), these data highlight the CDFs as potential breeding tools for improving the sink strength of the harvested potato organs in the *Solanaceae*. Furthermore, the higher yields promoted by CDFs were obtained under the highly heterogeneous field conditions.

## Data availability statement

The data presented in the study are deposited in the ProteomeXchange repository, accession number PXD036489.

## Author contributions

EB-F, JV-C, SGN, RVM and JM designed, planned, and organized the experiments. LC, EB-F, BR-M, FJM, JC, SC, LY, AS-L and MGC performed the research. SGN, RVM, JP-R and JM wrote the article. All authors contributed to the article and approved the submitted version.
