# Supplementary figures and images for: Ectopic expression of the AtCDF1 transcription factor in potato enhances tuber starch and amino acid contents and yield under open field conditions
Source: Front Plant Sci. 2023 Mar 1;14:1010669. doi: 10.3389/fpls.2023.1010669 (PMC10014720; doi:10.3389/fpls.2023.1010669)

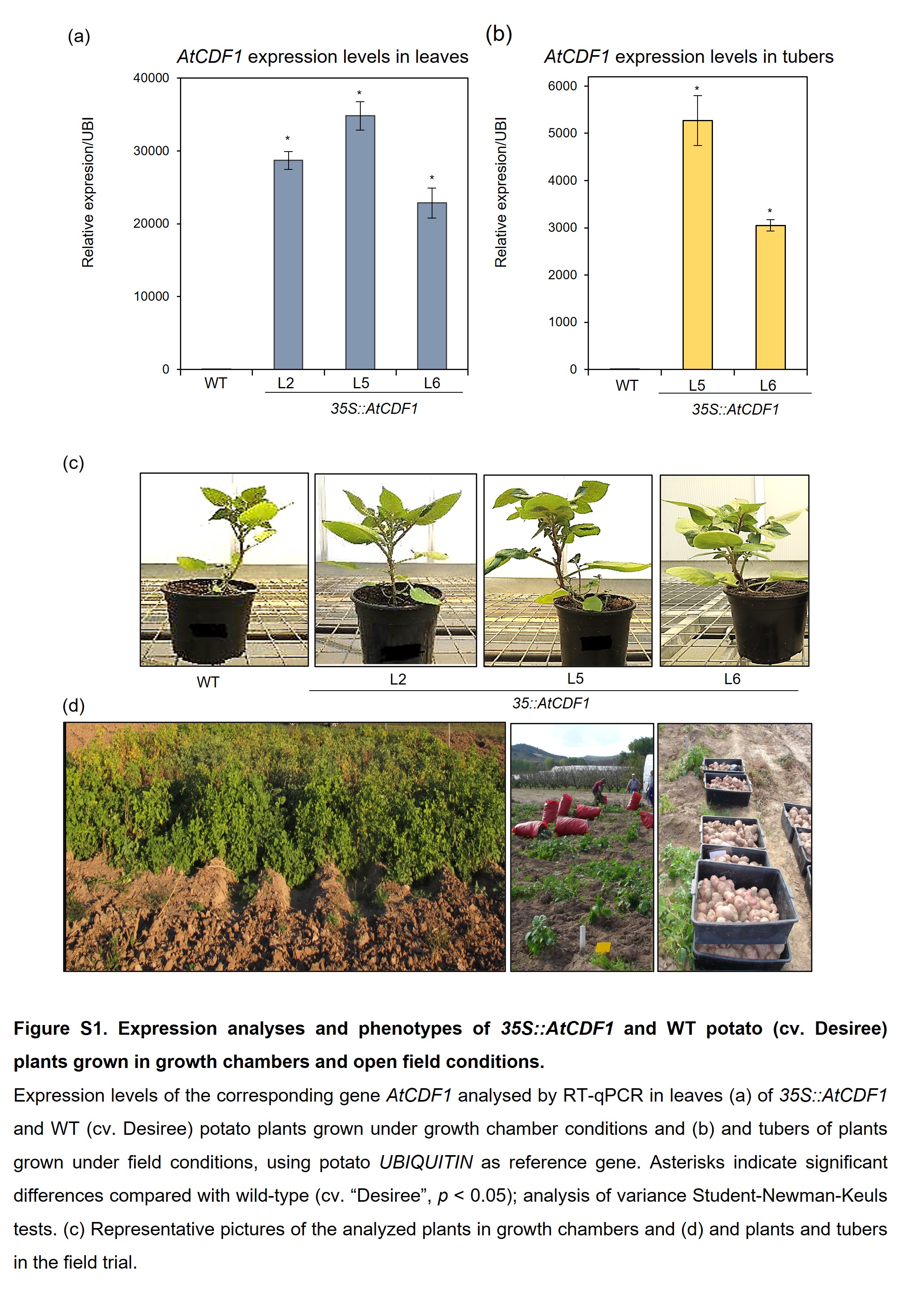

Supplement: Supplementary file 1 [file Image_1.jpeg]

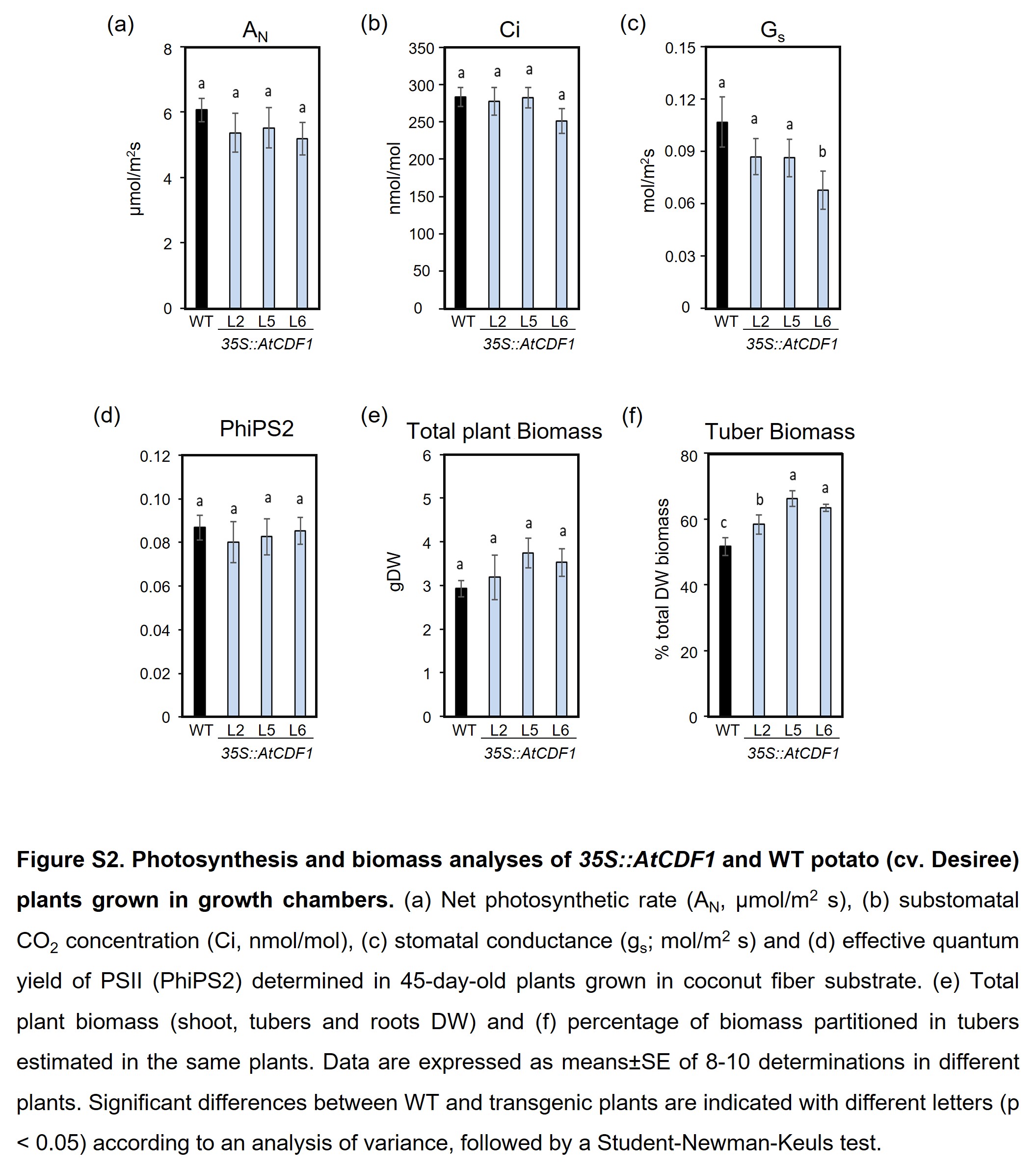

Supplement: Supplementary file 2 [file Image_2.jpeg]

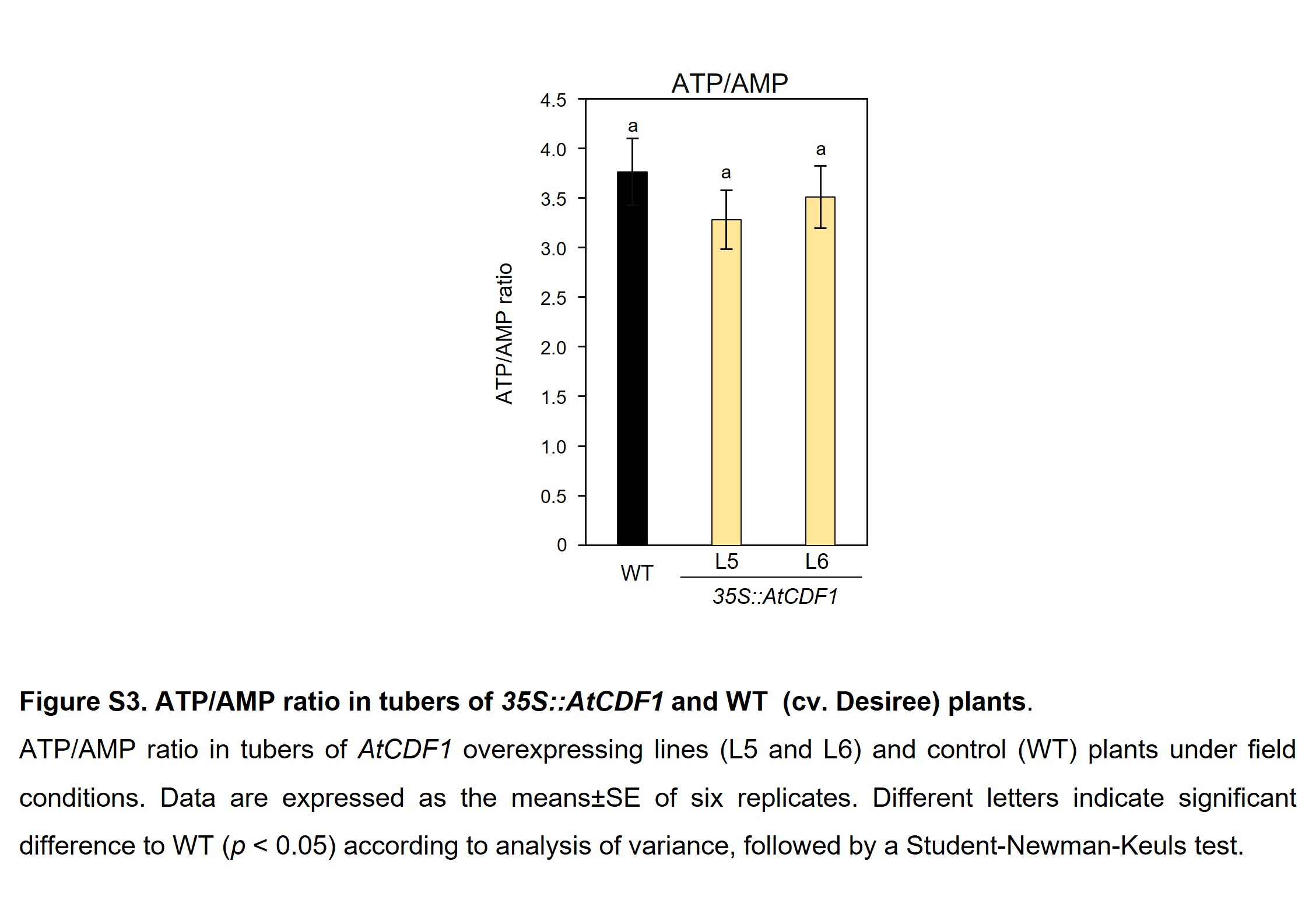

Supplement: Supplementary file 3 [file Image_3.jpeg]

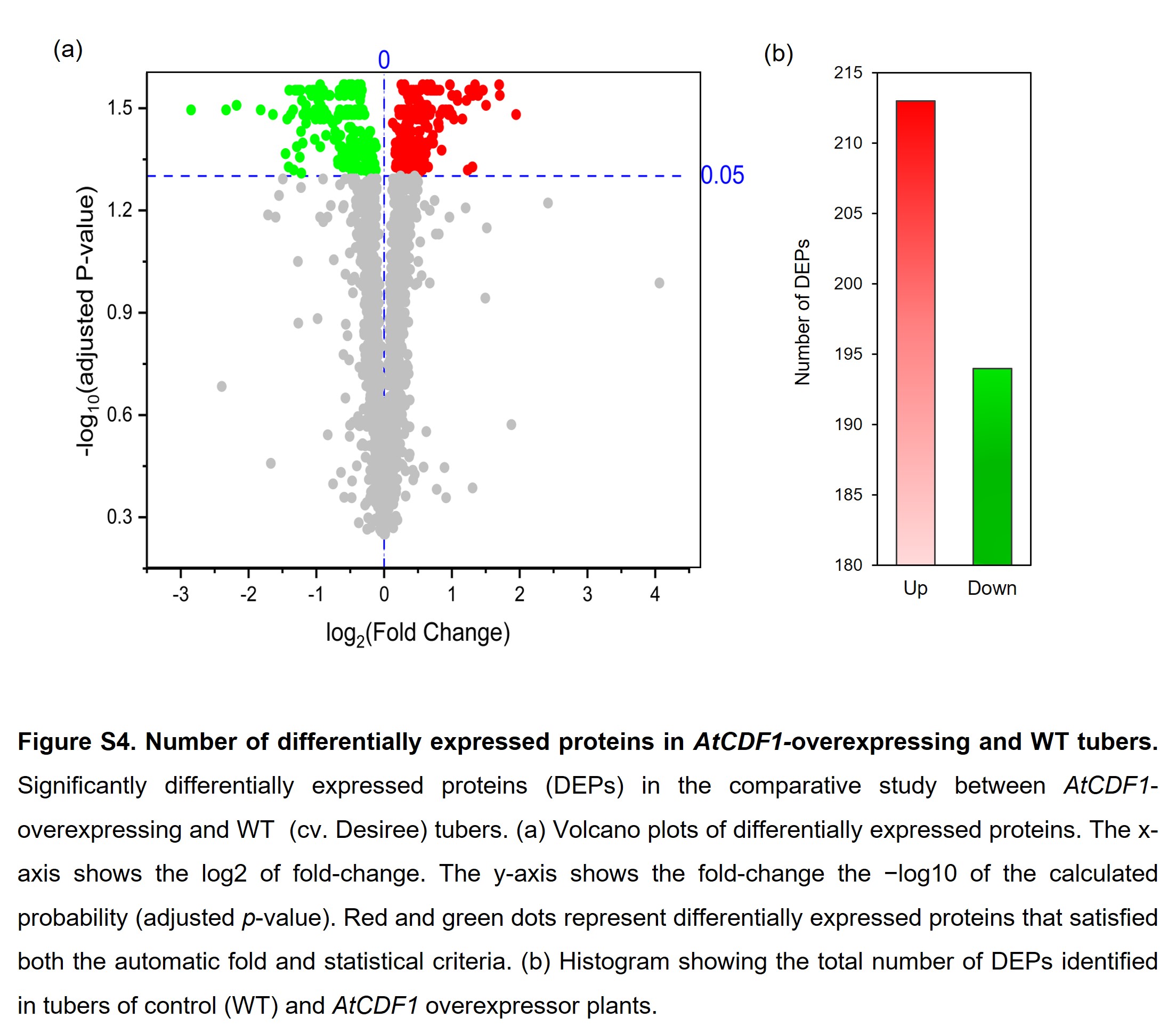

Supplement: Supplementary file 4 [file Image_4.jpeg]

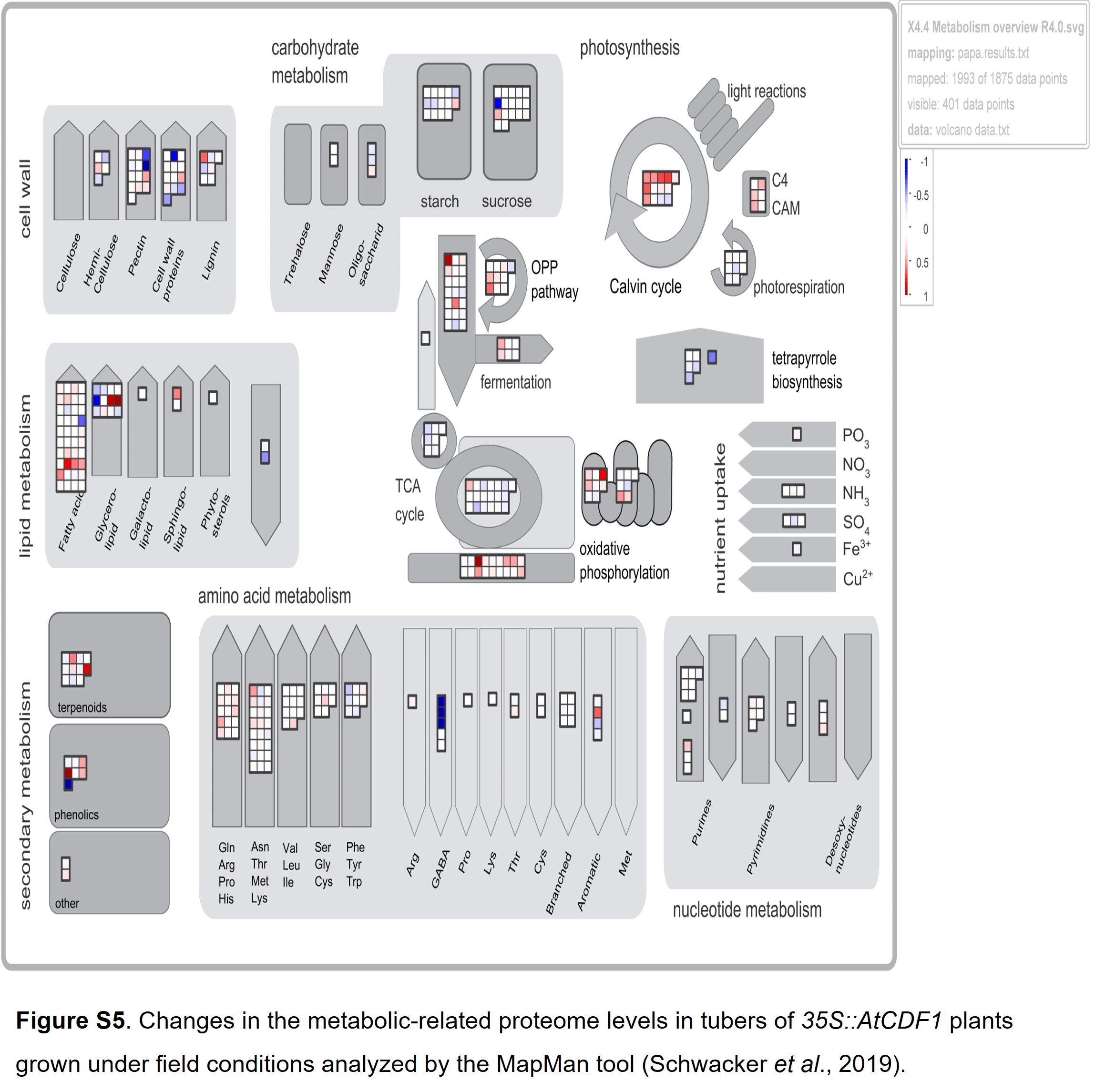

Supplement: Supplementary file 5 [file Image_5.jpeg]

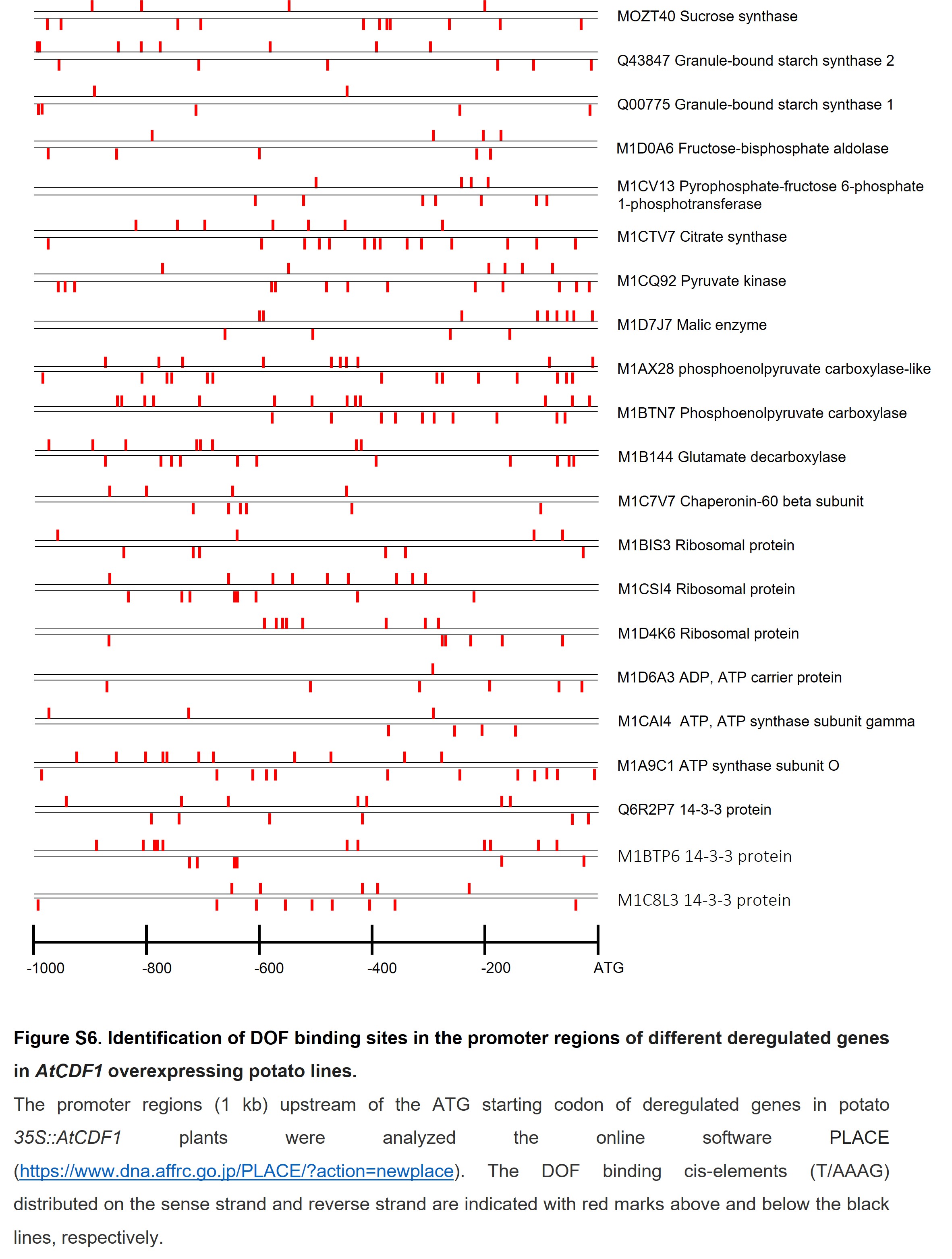

Supplement: Supplementary file 6 [file Image_6.jpeg]
